# Postural orthostatic tachycardia syndrome: when dysautonomia misleads: a mechanistic argument for compensatory orthostatic tachycardia

**DOI:** 10.3389/fneur.2026.1806502

**Published:** 2026-04-10

**Authors:** Pradeep Chopra

**Affiliations:** Warren Alpert Medical School of Brown University, Providence, RI, United States

**Keywords:** autoimmunity, autonomic failure, craniocervical instability, dysautonomia, hypermobile Ehlers-Danlos syndrome, hypovolemia, orthostatic intolerance, postural orthostatic tachycardia syndrome

## Abstract

Postural orthostatic tachycardia syndrome (POTS) is defined by chronic orthostatic intolerance accompanied by an excessive increment in sinus heart rate on standing in the absence of significant orthostatic hypotension. Contemporary reviews and consensus statements appropriately frame POTS as involving autonomic regulation. Yet the bedside phenotype most often observed-marked tachycardia with preserved blood pressure-also supports a hemodynamic interpretation: an intact baroreflex driving tachycardia to defend cardiac output and cerebral perfusion when effective stroke volume or venous return is reduced. This narrative, hypothesis-driven review argues that the umbrella label ‘dysautonomia’ is frequently applied imprecisely in POTS and is often interpreted (by clinicians and patients) as autonomic failure. In many patients, the dominant physiology is not autonomic failure but compensatory activation in response to orthostatic stressors such as low central blood volume, venous pooling, impaired vasoconstriction, or deconditioning. When the label is over-interpreted, management can drift toward reflexive heart-rate suppression rather than mechanism-directed evaluation and treatment of the orthostatic stressor. We propose a pragmatic, cardiology-facing framework that (1) distinguishes compensatory orthostatic tachycardia from primary autonomic failure (neurogenic orthostatic hypotension), and (2) prioritizes mechanistic phenotyping within POTS (low-preload/pooling dominant, neuropathic, hyperadrenergic, immune-associated, and secondary structural/CSF-pressure contributors). This reframing does not minimize the severity of POTS; rather, it supports clearer counseling and more targeted therapy by treating tachycardia as a signal of orthostatic stress and asking what is driving it.

## Highlights

- POTS is defined by orthostatic tachycardia with symptoms and without significant orthostatic hypotension; it is not synonymous with autonomic failure.
- Tachycardia with preserved BP is compatible with an intact baroreflex compensating for reduced stroke volume/venous return or impaired peripheral vasoconstriction.
- The term ‘dysautonomia’ is often over-interpreted as ‘autonomic failure,’ or ‘dysfunction of the autonomic nervous system’ which can misdirect counseling and treatment; precision matters.
- Mechanistic phenotyping enables targeted therapy and avoids reflexive heart-rate suppression in low-preload/pooling-dominant presentations.

## 1 Introduction

POTS is commonly defined as (i) frequent symptoms of orthostatic intolerance, (ii) a sustained increase in heart rate (HR) of at least 30 beats/min within 10 min of standing (≥40 beats/min in adolescents), and (iii) absence of significant orthostatic hypotension (blood pressure [BP] drop ≥20/10 mm Hg) (1–3).

Despite consistent diagnostic criteria, POTS is mechanistically heterogeneous. Major reviews emphasize roles for hypovolemia, venous pooling, deconditioning, peripheral autonomic neuropathy, hyperadrenergic states, and immune-mediated mechanisms. Consensus discussions highlight the need for individualized, mechanism-based management rather than a single unifying pathogenesis (4, 5).

POTS is frequently grouped under the label ‘dysautonomia.’ While this term can be clinically useful as shorthand for disorders involving autonomic regulation, it becomes problematic when interpreted as synonymous with autonomic failure or a ‘broken’ autonomic nervous system (ANS). The typical POTS bedside profile-marked tachycardia with near-preserved BP-differs fundamentally from neurogenic orthostatic hypotension, in which autonomic failure produces inadequate vasoconstriction and BP collapse.

## 2 Scope and method

This article is a narrative, hypothesis-driven review intended for a cardiology audience. We synthesize evidence from consensus statements and peer-reviewed reviews regarding POTS definitions and mechanisms, and we re-interpret the common clinical phenotype through a hemodynamic lens: orthostatic tachycardia as an output that may be expected (compensatory) or excessive relative to the underlying orthostatic stressor.

We do not argue that POTS is benign or ‘normal,’ nor do we claim that autonomic abnormalities are absent in all cases. Rather, we argue that the broad label ‘dysautonomia’ is frequently over-read as primary autonomic failure, and that reframing POTS as a compensatory syndrome improves mechanistic evaluation, patient counseling, and therapy selection.

## 3 Definitions: dysautonomia, autonomic failure, and POTS

In clinical practice, ‘dysautonomia’ is used variably to denote any disorder in which autonomic regulation contributes to symptoms. That broad usage is defensible but imprecise. Autonomic failure, by contrast, denotes impaired autonomic efferent function such that appropriate physiologic responses cannot be generated-classically producing neurogenic orthostatic hypotension with inadequate vasoconstrictor responses.

POTS generally does not meet the hemodynamic definition of orthostatic hypotension. Instead, many patients maintain BP while HR rises substantially. Preserved BP does not prove normal autonomic physiology, but it does argue against equating POTS with autonomic failure and supports the inference that key reflex arcs can function. The system is responding to a sensed reduction in effective stroke volume/venous return or vascular tone by increasing HR to defend cardiac output and cerebral perfusion (6).

Thus, the clinically useful distinction is not whether the ANS is involved-it almost certainly is-but whether the dominant clinical problem is autonomic failure versus compensatory activation in response to an orthostatic stressor.

## 4 Orthostatic tachycardia as compensation: a hemodynamic argument

Upon standing, gravitational translocation of blood to the lower body and splanchnic capacitance reduces venous return, preload, and stroke volume. In healthy individuals, baroreflex-mediated sympathetic activation produces modest tachycardia and vasoconstriction to preserve arterial pressure and cerebral blood flow.

In many POTS patients, the orthostatic reduction in effective preload appears exaggerated due to one or more of: (i) low circulating volume, (ii) excessive venous capacitance and pooling (especially splanchnic and lower extremity), (iii) impaired peripheral vasoconstriction (neuropathic or pharmacologic), and/or (iv) deconditioning with reduced stroke-volume reserve. Under these conditions, a larger HR increment is a predictable compensatory response rather than proof of a primary ‘overactive’ autonomic disorder.

This interpretation aligns with the observation that many patients experience orthostatic symptoms without frank hypotension: tachycardia is one of the principal defenses against syncope. Symptoms-palpitations, tremulousness, fatigue, cognitive slowing-can be understood as the physiologic cost of sustaining high sympathetic drive to maintain perfusion.

## 5 Mechanistic phenotypes supporting a compensatory framework

### 5.1 Low preload: hypovolemia and deconditioning

Relative hypovolemia and physical deconditioning are common in POTS. Reduced plasma volume and/or impaired renin-angiotensin-aldosterone responses can lower baseline preload. Deconditioning (often secondary to symptom-driven inactivity) reduces stroke volume, magnifying the HR rise required to maintain cardiac output during orthostasis.

These mechanisms fit the compensatory model: the ANS increases HR because stroke volume cannot be maintained. Clinical improvement with salt/fluid loading, structured recumbent exercise reconditioning, and compression strategies supports the view that restoring preload and venous return can reduce tachycardia and symptoms.

### 5.2 Venous pooling and connective tissue laxity

Excessive venous pooling is a recurrent theme in POTS pathophysiology. Increased venous compliance and impaired connective tissue support may augment venous capacitance in the lower extremities and pelvis/splanchnic bed, amplifying orthostatic reductions in venous return.

Cross-sectional data show that a substantial minority of POTS patients meet clinical criteria for hypermobile Ehlers-Danlos syndrome (hEDS) and many more exhibit generalized joint hypermobility. This overlap is clinically relevant because it increases the prior probability of pooling-dominant physiology and supports early use of lower-body and abdominal compression, counter-maneuvers, and volume expansion (7).

### 5.3 Neuropathic POTS and partial autonomic impairment

Some patients demonstrate evidence of small-fiber neuropathy or impaired sympathetic vasoconstriction in dependent vascular beds. Even when neuropathic mechanisms are present, the observed tachycardia can be interpreted as compensatory: if peripheral vasoconstriction is inadequate, HR increases to defend pressure and flow.

This nuance matters. Neuropathic contributions may justify inclusion of POTS under a broad dysautonomia umbrella, but they do not imply autonomic failure in the same way as neurogenic orthostatic hypotension. Clinically, the compensatory framework still guides therapy toward improving venous return and vascular support while using medications selectively and phenotype-driven.

### 5.4 Hyperadrenergic POTS

Hyperadrenergic POTS is characterized by excessive sympathetic activation and, in some patients, orthostatic hypertension or elevated standing norepinephrine. This phenotype is sometimes presented as evidence against a compensatory model. However, exaggerated sympathetic output may be a downstream response to pooling/low preload, altered baroreflex gain, central autonomic network dysregulation, or genetic susceptibility.

Accordingly, the present argument is not that sympathetic activation is always ‘appropriate,’ but that it is often downstream of an orthostatic stressor and should be interpreted in that context rather than treated as the primary disease in every patient.

### 5.5 Immune-mediated mechanisms

A subset of patients have autoimmune comorbidity and, in some cohorts, circulating autoantibodies directed at autonomic receptors. Anti-ganglionic acetylcholine receptor antibodies have been reported in a minority of patients, and other studies report functional adrenergic and muscarinic receptor antibodies (5, 8).

These findings support immune-mediated perturbations of autonomic signaling in selected patients. Yet orthostatic tachycardia can still represent a compensatory final common pathway: immune-mediated vasomotor impairment or receptor-level signaling changes reduce effective vascular resistance or venous return, and HR rises to preserve perfusion. Because immunotherapy data are limited and heterogeneous, causal language should remain cautious; however, identifying and treating coexisting autoimmune disease may still improve symptoms in selected patients (9).

### 5.6 Secondary structural/CSF-pressure contributors

Orthostatic intolerance and POTS-like phenotypes have been described in association with craniocervical junction disorders (e.g., Chiari I malformation, craniocervical/upper cervical instability) and CSF-pressure disorders (especially spontaneous intracranial hypotension) (6, 10–15). These associations are biologically plausible because key autonomic nuclei and pathways including the nucleus tractus solitarius (NTS), dorsal motor nucleus of the vagus, and rostral ventrolateral medulla (RVLM) reside within the medulla and participate in baroreflex integration and sympathetic outflow control (16, 17).

For cardiology readers, this literature warrants disciplined interpretation: many reports are observational, involve highly selected referral cohorts, and do not exclude coexisting low-preload physiology, deconditioning, or hyperadrenergic traits (12, 18). Nonetheless, the collective data support a secondary-cause mindset, particluarly when orthostatic symptoms co-occur with occipital/valsalva headaches, cranial neuropathic features, focal neurologic signs, cough syncope, or orthostatic headache suggestive of CSF-pressure abnormalities (12, 13, 15, 19). In such cases, targeted neuroimaging (brain/cervicomedullary MRI cine CSF flow, and/or evaluation for CSF leak) and multidisciplinary consultation may be reasonable (12, 15).

Mechanistic pathways that could yield a POTS-like phenotype include:

Impaired baroreflex afferent integration at the NTS (or its connections), with maladaptive sympathetic activation during orthostatic stress and disproportionate HR rise despite preserved (or labile) BP (16, 17).
Disruption of medullary cardioinhibitory/vagal nuclei or descending autonomic pathways, reducing effective parasympathetic restraint and increasing reliance on compensatory tachycardia to maintain cardiac output and perfusion pressure (12, 16).
Mechanical compression/traction at the cervicomedullary junction (e.g., tonsillar ectopia, basilar invagination, instability-related deformation) producing episodic brainstem dysfunction that manifests as orthostatic tachycardia, presyncope/syncope, and other orthostatic intolerance symptoms (12–14, 19).
CSF-pressure disorders (particularly CSF leak syndromes) lowering intracranial CSF volume and triggering orthostatic symptoms; in select cases, treatment of the leak (e.g., epidural blood patch) improves orthostatic tachycardia, supporting secondary POTS (15).

## 6 A proposed clinical framework: from label-based to mechanism-based care

Treat ‘POTS’ as a syndrome requiring mechanistic phenotyping rather than as a monolithic dysautonomia. Reserve ‘autonomic failure’ for neurogenic orthostatic hypotension phenotypes. Use ‘POTS’ to describe the orthostatic tachycardia syndrome, then specify the dominant mechanism(s) when possible.

A mechanism-first approach improves patient counseling and guides therapy. A practical explanation is: ‘Your heart rate is rising to defend blood flow when upright; our job is to identify what is driving the orthostatic stress and treat that driver.’ This preserves the core argument: tachycardia is often not the primary disease, and reflexive HR suppression may be counterproductive in low-preload/pooling phenotypes.

## 7 Implications for cardiology practice

Confirm diagnostic criteria and exclude mimics (anemia, thyroid disease, dehydration, medication effects, inappropriate sinus tachycardia).

Prioritize nonpharmacologic therapies that improve preload and venous return (salt/fluid loading, lower-body and abdominal compression, graded recumbent exercise reconditioning) as first-line management.

Select pharmacologic therapy based on phenotype: rate control (low-dose beta-blocker or ivabradine) may help some; vasoconstrictors and volume expanders may be prioritized in pooling/hypovolemia; centrally acting sympatholytics may be considered selectively in hyperadrenergic phenotypes.

Maintain vigilance for secondary causes (connective tissue disorders, immune-mediated disease, and selected structural/CSF-pressure syndromes) when symptoms are atypical or refractory.

## 8 Limitations of the argument

Preserved BP does not exclude autonomic dysfunction; it argues against equating POTS with autonomic failure. Mechanistic categories overlap and many patients have mixed physiology. Evidence supporting some secondary structural contributors is limited by referral bias and study design. Finally, the term ‘dysautonomia’ is not necessarily wrong in a broad sense; the critique is directed at its frequent over-interpretation as implying autonomic failure and at the clinical consequences of that misunderstanding.

## 9 Conclusion

POTS is a heterogeneous orthostatic intolerance syndrome with a shared clinical definition but multiple pathophysiologic drivers. In many patients, tachycardia with preserved BP is congruent with an intact reflex arc compensating for orthostatic stress (low preload, pooling, or impaired vasoconstriction).

Using ‘dysautonomia’ as a blanket explanation can mislead clinicians and patients by implying autonomic failure and encouraging reflexive heart-rate suppression. A mechanism-first framework-distinguishing compensatory orthostatic tachycardia from autonomic failure and then phenotyping the dominant driver-supports more precise evaluation, clearer counseling, and targeted therapy.
